# Structural and biochemical characterization of the C‐terminal region of the human RTEL1 helicase

**DOI:** 10.1002/pro.5093

**Published:** 2024-08-24

**Authors:** Giuseppe Cortone, Melissa A. Graewert, Manil Kanade, Antonio Longo, Raghurama Hegde, Amaia González‐Magaña, Belén Chaves‐Arquero, Francisco J. Blanco, Luisa M. R. Napolitano, Silvia Onesti

**Affiliations:** ^1^ Structural Biology Laboratory Elettra‐Sincrotrone Trieste Trieste Italy; ^2^ International School for Advanced Studies (SISSA) Trieste Italy; ^3^ European Molecular Biology Laboratory (EMBL) Hamburg Hamburg Germany; ^4^ Department of Chemistry Università degli Studi di Trieste Trieste Italy; ^5^ Instituto Biofisika and Departamento de Bioquímica y Biología Molecular (CSIC, UPV/EHU) University of the Basque Country Leioa Spain; ^6^ Centro de Investigaciones Biológicas Margarita Salas (CIB) CSIC Madrid Spain

**Keywords:** DNA repair and replication, G‐quadruplexes, harmonin homology domain, intrinsically disordered proteins (IDP), PCNA‐binding peptide (PIP), protein‐nucleic acid interaction, R‐loops and D‐loops, structural biology, telomere maintenance

## Abstract

RTEL1 is an essential DNA helicase which plays an important role in various aspects of genome stability, from telomere metabolism to DNA replication, repair and recombination. RTEL1 has been implicated in a number of genetic diseases and cancer development, including glioma, breast, lung and gastrointestinal tumors. RTEL1 is a FeS helicase but, in addition to the helicase core, it comprises a long C‐terminal region which includes a number of folded domains connected by intrinsically disordered loops and mediates RTEL1 interaction with factors involved in pivotal cellular pathways. However, information on the architecture and the function of this region is still limited. We expressed and purified a variety of fragments encompassing the folded domains and the unstructured regions. We determined the crystal structure of the second repeat, confirming that it has a fold similar to the harmonin homology domains. SAXS data provide low‐resolution information on all the fragments and suggest that the presence of the RING domain affects the overall architecture of the C‐terminal region, making the structure significantly more compact. NMR data provide experimental information on the interaction between PCNA and the RTEL1 C‐terminal region, revealing a putative low‐affinity additional site of interaction. A biochemical analysis shows that the C‐terminal region, in addition to a preference for telomeric RNA and DNA G‐quadruplexes, has a high affinity for R‐loops and D‐loops, consistent with the role played by the RTEL1 helicase in homologous recombination, telomere maintenance and preventing replication‐transcription conflicts. We further dissected the contribution of each domain in binding different substrates.

## 1 INTRODUCTION

RTEL1 (regulator of telomere length 1) is a DNA helicase which belongs to the iron–sulfur (FeS) cluster helicase family, together with the human paralogues XPD (Xeroderma pigmentosum complementation group D), FANCJ (Fanconi's anemia complementation group J), and DDX11 helicase (DEAD/H‐box helicase 11) (Wu & Brosh, 2012).

RTEL1 was initially identified as a key factor involved in the stability and elongation of telomeres, but was then shown to be important in both DNA replication and repair (Hourvitz et al., 2024; Uringa et al., 2012; Vannier et al., 2014). Moreover, RTEL1 was shown to be important for the stability of fragile sites, and is involved in mitotic DNA synthesis (MiDAS) at regions prone to form G‐quadruplexes/R‐loops structures (Wu et al., 2020). Deletion of the helicase causes R‐loop accumulation and global transcriptional changes suggesting that the replication defects observed in *Rtel1*‐deficient cells could be due to an inefficient removal of G4/R‐loops (Björkman et al., 2020; Kotsantis et al., 2020; Wu et al., 2020). Although RTEL1 has been reported to resolve D‐loops and G4 in vitro (Vannier et al., 2013; Youds et al., 2010) and to bind Telomeric Repeats containing RNAs (TERRAs) with high affinity (Ghisays et al., 2021), the exact role of RTEL1 in DNA replication, DNA repair and telomere maintenance is still not completely clarified.

Several mutations in the human *Rtel1* gene have been associated to genetic diseases, such as familial Pulmonary Fibrosis and Hoyeraal‐Hreidarsson syndrome, a severe form of Dyskeratosis Congenita characterized by accelerated telomere shortening and a multisystem bone‐marrow failure (Ballew et al., 2013; Deng et al., 2013; LeGuen et al., 2013). *Rtel1* gene amplification has been observed in gastrointestinal cancer and adrenocortical carcinoma (Bai et al., 2000; Yuan et al., 2022) and *Rtel1* single‐nucleotide polymorphisms have been associated with increased susceptibility to glioma (Melin et al., 2017; Namgoong et al., 2018; Shete et al., 2009; Wrensch et al., 2009; Wu et al., 2019) underlying a key role for RTEL1 in cancer development (Hassani et al., 2023).

The architecture of RTEL1 encompasses a catalytic domain that is expected to fold as a canonical helicase belonging to the FeS helicase family. In addition, the protein includes a C‐terminal domain, having different lengths in various orthologues, and comprising multiple domains which are likely to mediate the interaction of RTEL1 with factors involved in a variety of cellular pathways. In the human sequence, two Harmonin Homology Domains (HHDs) have been predicted (Faure et al., 2014). HHD1 was shown to mediate the interaction of RTEL1 with SLX4 to ensure smooth DNA replication in unstressed cells and prevent replication‐transcription conflicts (Takedachi et al., 2020). SLX4 is a scaffold protein that is implicated in a variety of DNA repair processes, including interstrand DNA crosslink repair, homologous recombination, telomere maintenance and the resolution of stalled replication forks. SLX4 was also shown to be required for RTEL1 recruitment to loci prone to form G4‐associated R‐loops (Wu et al., 2020). The HHD2 domain has been implicated in the interaction with Replication Protein A (RPA), the single‐stranded DNA binding protein that has a key function in most DNA replication, DNA repair, DNA damage signaling pathways.

Another important interaction partner is Proliferating Cell Nuclear Antigen (PCNA), the DNA clamp that acts as processivity factor for DNA polymerases in DNA replication. A sequence of eight amino acids following the second HHD was identified as a canonical PCNA‐interaction‐protein box (PIP‐box) responsible for RTEL1 interaction with the replisome via PCNA; mutation of the RTEL1 PIP‐box in mice exhibited accelerated tumor formation, increased predisposition to medulloblastoma, and decreased survival (Vannier et al., 2013). At the very end of RTEL1, there is a putative C_4_C_4_ RING finger domain, that is involved in the interaction with TRF2, a component of the Shelterin complex, to recruit RTEL1 to telomeres and promote T‐loop disassembly (Sarek et al., 2015).

Despite the importance of the RTEL1 C‐terminal region as an interaction hub with a plethora of factors that are important in genome maintenance, a comprehensive analysis of this region is lacking; moreover the contribution of each domain to the selection of the helicase substrate has not been addressed. Here, we present a comprehensive structural and biochemical study of the organization of the C‐terminal region of human RTEL1. Our bioinformatics analysis confirms that this region presents conserved domains with a globular fold, interspersed with low complexity sequences, and highlights the evolution of the C‐terminal region in RTEL1 orthologues. We expressed and purified a number of fragments encompassing various domains and analyzed them by structural and biochemical methods. We determined the crystal structure of the second folded repeat and confirmed that it indeed folds as an HHD. We used Small‐Angle X‐ray Scattering (SAXS) to obtain structural information on the architecture of all the fragments: SAXS data reveal that the C‐terminal region without the RING domain is highly flexible, while when the RING domain is present, it has a more compact shape. Finally, by performing a comprehensive biochemical analysis, we also show that the whole RTEL1 C‐terminal domain preferentially binds with high‐affinity TERRA G4, D‐loop and R‐loop substrates. By testing the affinity of each fragment, we were able to dissect the role of the folded domains and linker regions in the interaction with various nucleic acid substrates. Our data provide a useful framework to elucidate the role of the RTEL1 C‐terminal domain at the interface between DNA recombination, repair and replication.

## 2 RESULTS AND DISCUSSION

### 2.1 Predicted architecture and evolution of the RTEL1 C‐terminal domains

**FIGURE 1:**
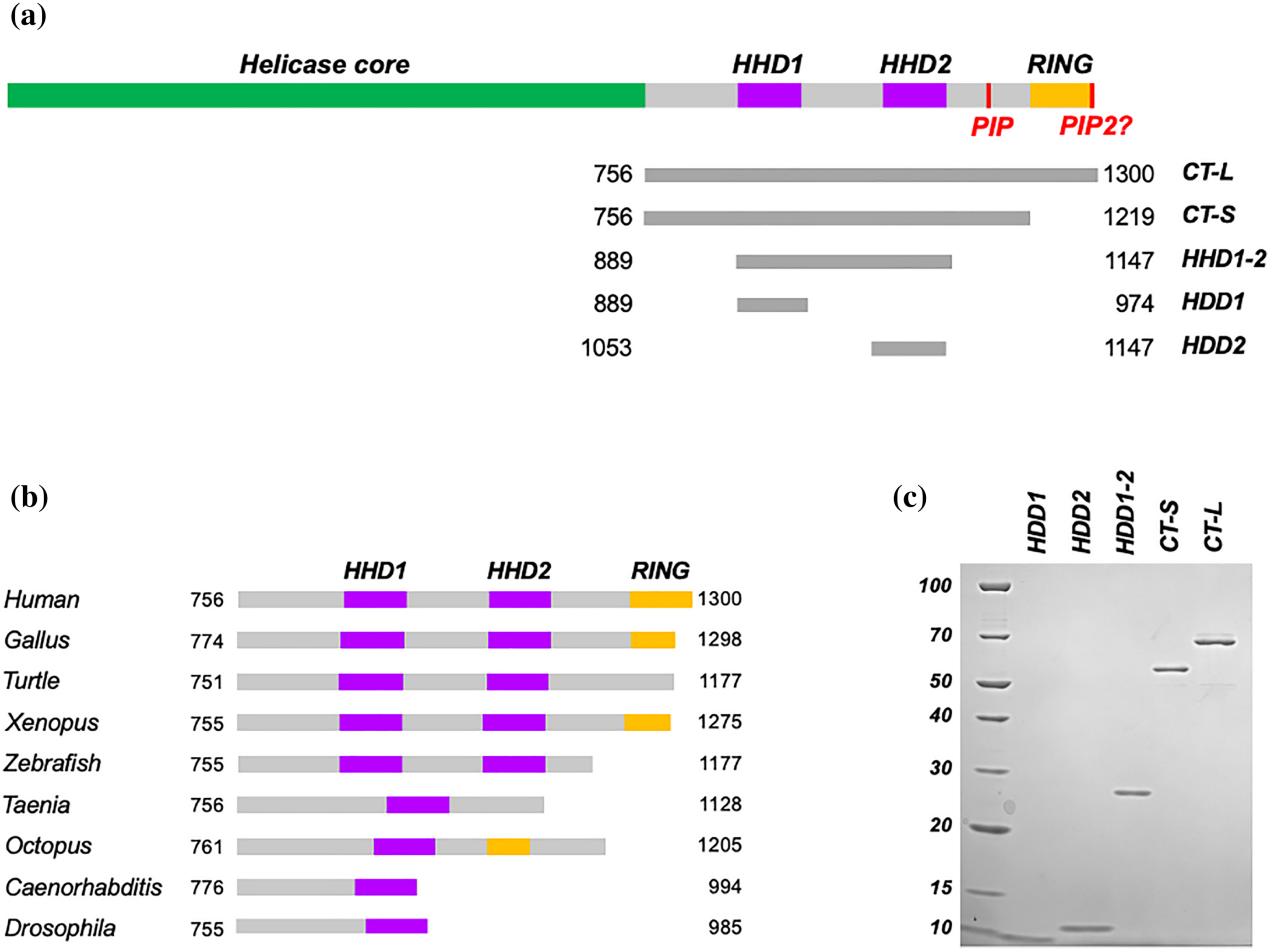
Dissection of hRTEL1 C‐terminal region. (a) A schematic diagram of the predicted architecture of human RTEL1; beside the helicase core (in green), the C‐terminal domain is constituted by regions that are expected to be unstructured (in gray), interspersed with folded domain, such as two Harmonin Homology domains (HHD, in magenta) and a RING finger domain (in yellow); a PCNA‐interacting peptide (PIP) box following the HHD2 has been described (Vannier et al., 2013), while a second putative PIP box can be identified at the C‐terminal end. Below a diagram showing the domain boundaries of the fragments which have been cloned, expressed and purified. (b) A schematic representation of the architecture of RTEL1 C‐terminal region for different species across evolution, color‐coded as in panel a. (c) SDS PAGE analysis of the purified hRTEL1 C‐terminal fragments.

Among the helicases belonging to the FeS helicase family, RTEL1 is characterized by the presence of a unique C‐terminal region, containing some folded domains connected by flexible links (Figure 1a). Alternative splicing generates multiple RTEL1 isoforms of different lengths: the most studied isoforms comprise 1219 and 1300 amino‐acid residues, and are identical throughout most of the sequence, with a difference at the C‐terminus.

In the core of the C‐terminal region, two structured domains were variably predicted to fold as HHD, TPR or HEAT repeats by a variety of prediction algorithms, with the more recent models based on artificial intelligence agreeing with the initial proposal of a HHD fold (Faure et al., 2014). HHD repeats are not very common and have only been found in a handful of proteins; all of these proteins, with the exception of RTEL1, are expressed in sensory neurons, and are involved in intricate networks mediating protein–protein interaction (Colcombet‐Cazenave et al., 2021). Interestingly, RTEL1 seems to be the only protein possessing two HHDs, rather than one. An analysis of the RTEL1 sequences shows that this duplication has occurred later in evolution: when comparing the sequences of orthologues from different orders/phyla, the presence of a second harmonin homology domain seems to occur in vertebrates, whereas Arthropoda, Mollusca, Nematoda, Platyhelminthes, Annelida all have a single HHD (Figure 1b).

Less clear is the evolutionary pattern for the C‐terminal RING finger domain. Although this domain is also absent in lower phyla, the evolutionary pattern seems to be more confusing, and it is more difficult to draw general conclusions. It must be stressed that human RTEL1 is present in multiple isoforms, derived from alternative splicing, and only the longer versions of the protein (1300 aa) contain the RING finger; it is possible that the confusing conservation pattern may be due to a poor characterization of the multiple isoforms in most species. However, a puzzling feature is the presence of a RING domain in Octopus.

### 2.2 Dissection and purification of human RTEL1 C‐terminal fragments

In order to perform a complete structural and biochemical characterization of the C‐terminal region of the human RTEL1 helicase, based on the bioinformatic analysis we identified the boundaries of each domain within the human sequence. Following this analysis, we designed a number of constructs to express and purify a number of fragments from bacterial cells: these include the entire C‐terminal region (C‐terminus long, CT‐L), the C‐terminal region lacking the final RING domain (C‐terminus short, CT‐S) and the single HHD1 and HHD2 domains (Figure 1a) to further investigate the structural and functional role of the linker region which connects HHD1 and HHD2, a fragment spanning both repeats was also designed (HHD1‐2). All the domains were purified by affinity chromatography, followed by ion exchange chromatography and size exclusion chromatography, in order to obtain pure and homogeneous samples (Figure 1c).

### 2.3 NMR and ITC analysis of PCNA binding to the human RTEL1 PIPs

A canonical PIP‐box (PCNA‐interaction‐protein box) was identified downstream the HHD2 domain, and experimental data confirmed that it could be responsible for the interaction between PCNA and RTEL1 (Vannier et al., 2013, 2014). Canonical PIP‐box motifs follow the pattern Qxxφxxψψ with φ being an aliphatic hydrophobic residue (L, M, I, V) and ψ an aromatic residue (most often Y or F), but a number of very divergent sequences have been observed (Prestel et al., 2019). A stretch of 12 amino acids at the very end of the C‐terminal domain, downstream the RING finger domain shows some similarity to the canonical PIP‐box domain.

**FIGURE 2:**
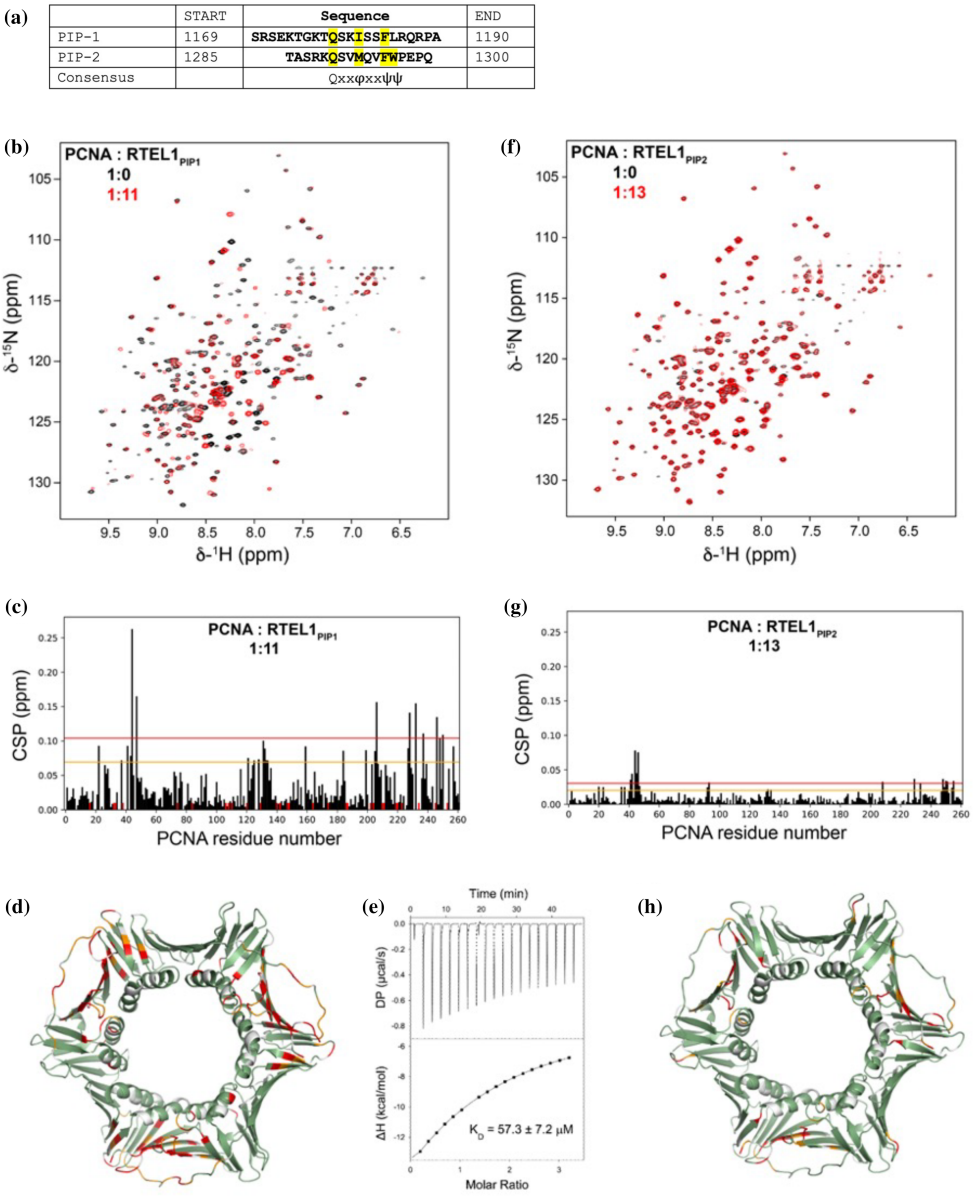
Analysis of the interaction between RTEL1 PIP boxes and PCNA. (a) Peptides corresponding to the canonical (PIP1) and the putative (PIP2) PIP‐box motifs of human RTEL1, compared with the canonical PIP‐box pattern Qxxφxxψψ, where φ is an aliphatic hydrophobic residue (L, M, I, V) and ψ an aromatic residue (Y, F). The RTEL1 residues that conform to the canonical pattern have been highlighted in yellow. (b) Overlay of the NMR ^1^H,^15^N TROSY spectra of PCNA in the absence (black) and in the presence (red) of a 11‐fold molar excess of RTEL1_PIP1_ peptide. (c) Bar graph of weighted chemical shift perturbations (CSPs) in the spectra of PCNA induced by the RTEL1_PIP1_ peptide as a function of residue number. The lines indicate the mean plus one (orange) or two (red) standard deviations. Red bars indicate residues for which the signals in the bound state could not be assigned (due to a very large CSP or signal disappearance). (d) PCNA residues that exhibit PIP1‐induced resonance changes are mapped onto the crystal structure of PCNA (PDB entry 1VYM) and colored red (CSP larger than average plus two standard deviations or signal disappearance) or orange (CSP larger than average plus one standard deviation). Residues with smaller CSPs are colored green, and those with no data available are colored gray. (e) ITC of RTEL1_PIP1_ peptide binding to PCNA. In the upper panel the heat change caused by peptide binding; in the lower panel the integration of each peak plotted against the molar ratio peptide:PCNA. The squares correspond to the experimental data and the continuous line corresponds to the best fit to a model of one set of identical binding sites. (f) Same as panel b for RTEL1_PIP2_ peptide. (g) Same as panel c for RTEL1_PIP2_ peptide. (h) Same as panel d for RTEL1_PIP2_ peptide.

To experimentally test this hypothesis, we studied two peptides (PIP‐1 and PIP‐2) encompassing the sequences of the canonical and putative PIP‐boxes (Figure 2a). We characterized the interaction of PCNA with PIP1 and PIP2 peptides in solution by NMR at 35°C, measuring the changes in the ^1^H‐^15^N‐correlation spectra of PCNA caused by the addition of the unlabeled peptides. The PIP1 peptide caused large shifts in many signals (Figure 2b,c), but no signal duplication, indicating a 1:1 stoichiometry. Mapping of signal changes on the crystal structure of PCNA identified the binding site to the region on each PCNA protomer where PIP sequences of other proteins are known to bind (Figure 2d). Indeed, calorimetry data showed a dissociation constant of 53.3 ± 7.2 μM (Figure 2e). In contrast, the PIP2 peptide caused much smaller shifts (Figure 2f,g), indicating a very low affinity binding, with a likely dissociation constant in the mM range (De Biasio et al., 2012). However, those residues experiencing significant changes mapped to the same site on the PCNA protomer (Figure 2h).

These results seem to point out that both PIP motifs bind to the same site on PCNA but with very different affinities. While the affinity of PIP1 is in the range of many other PIP sequences (González‐Magaña & Blanco, 2020), PIP2 shows a much lower affinity for PCNA raising the doubt it may not be physiologically relevant. However, the presence of multiple PIP boxes with a wide range of affinities is not unusual, and it may contribute to increase the avidity of RTEL1 for PCNA, or to play a role in a dynamic network of interactions. A similar situation was shown to occur for the F‐box DNA helicase, which includes two motifs binding to PCNA with different affinities, or for a number of DNA polymerases and ligases which also seem to systematically include multiple PIP boxes (Hamdan & De Biasio, 2023; Masuda et al., 2015). Cellular, biochemical and structural data seem to confirm that a number of proteins use multiple, canonical and non‐canonical PIP boxes displaying a range of affinities, which are involved in a complex and shifting interacting network (Hamdan & De Biasio, 2023).

### 2.4 Crystal structure of RTEL1 Harmonin homology domain 2

**FIGURE 3:**
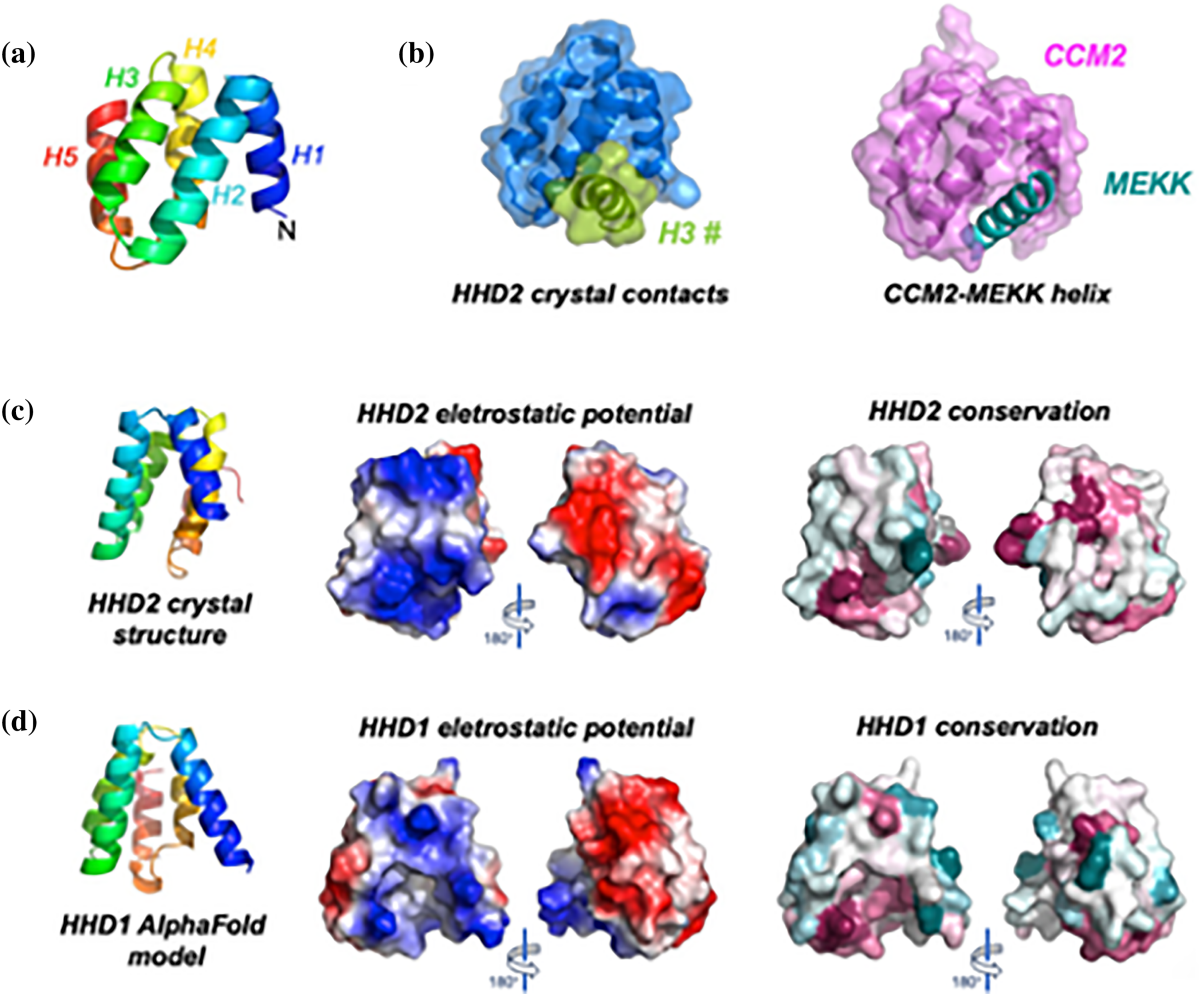
HHD2 crystal structure. (a) A rainbow representation of the HHD2 crystal structure, with the chain colored from blue to red, starting from the N‐terminus. (b) Within the crystal lattice, the molecules are arranged so that the groove between H1 and H2 interacts with H3 from a neighboring molecule (in green). (c) The complex between the HHD from CCM2 and the amphipathic helix from MEKK3 (PDB ID: 4YKC, Wang et al., 2015), showing the canonical interaction between HHD and an amphipatic helix. (c) The HHD2 crystal structure visualized perpendicularly to the groove between helices H1 and H2. The electrostatic surface potential (negative potential in red and positive potential in blue) shows a highly asymmetric charge distribution, with the “front” face highly positively charged, and the “back” face highly negatively charged. On the right panels the level of surface conservation is displayed so that highly conserved residues are in maroon, and no‐conserved residues in turquoise. (d) The same analysis has been carried out on HHD1, using the model provided by the AlphaFold server (Jumper et al., 2021). A similar pattern of charge distribution and sequence conservation is observed; it is interesting to notice that whereas HHD1 shows a similar “groove” as the other harmonin homology folds (i.e. CCM2), in HHD2 the groove is less pronounced; indeed the orientation of the neighboring helix (H3#) that is interacting with H1 and H2 has a different orientation from the canonical amphipathic helices. For both HHD1 and HHD2 further details on the conserved residues are shown in Figure S2.

The human C‐terminal domain contains two predicted harmonin homology domains (HHDs). We obtained crystals of the human HHD2 that diffracted to 2.3 Å resolution (Table 1); the structure was determined using the AlphaFold (Jumper et al., 2021) prediction as a search model for Molecular Replacement, trimming the N‐terminal helix. The final model conforms to the canonical fold of a HHD, folding as a 5‐helices bundle, with an up‐down‐up‐down‐up topology, and with the first helix less tightly packed against the rest of the bundle (Figure 3a). The same structure was recently independently determined (Kumar et al., 2024). The seven molecules in the asymmetric unit are all virtually identical, with at most a difference of one or two residues at the N or C‐termini. In the crystal lattice, the molecules are arranged in a pattern so that the groove between H1 and H2 accommodates Helix 3 from a neighboring molecule (Figure 3b).

**TABLE 1:** Crystallographic data collection and refinement statistics for HHD2 domain.

PDB entry	8P8H
Source	Elettra XRD2/11.2C
Wavelength (Å)	1.0
Resolution limits (Å)	50–2.3
Space group	*P2*_*1*_
Unit‐cell parameters	*a* = 65.59 Å *b* = 60.68 Å *c* = 79.53 Å *β* = 96.13°
No. of observations	181,386
No. of unique reflections	27,255
Redundancy	6.0
Completeness (%)	97.9 (96.2)
Mean *I*/*σ* (*I*)	10.53 (1.99)
*R*_merge_ ^a^	0.14 (0.98)
CC_1/2_ ^b^	0.997 (0.836)
Refinement statistics	
Resolution limits (Å)	48.14–2.3
No. of reflections	25,886 (1866)
Protein/water atoms	4222/32
*R*_cryst_ ^c^	0.21 (0.30)
*R*_free_ (5% of data)	0.279 (0.330)
RMSD bonds (Å)	0.01
RMSD angles (°)	1.58
<*B*> factor (Å^b^)	46.17
Ramachandran plot analysis (%) favored/allowed/outliers	97.43/1.29/1.29

*Note*: Values in parentheses indicate statistics for the high–resolution data bin for X‐ray and refinement data.

^a^ *R*_merge_ = ∑*hkl* ∑*i* |*I* (*hkl*) *i* − <*I* (*hkl*)>|/∑*hkl* ∑*i* <*I* (*hkl*)*i*>.

^b^ CC_1/2_ is the correlation coefficient between two random half datasets.

^c^ *R*_cryst_ = ∑*hkl* |*F*_o_ (*hkl*) − *F*_c_ (*hkl*)|/∑*hkl* |*F*_o_ (*hkl*)|, where *F*_o_ and *F*_c_ are observed and calculated structure factors, respectively.

An approximate calculation of the electrostatic potential for the HHD2 crystal structure was carried out and displayed using PyMol (Schrodinger, 2015). The charge distribution appears to be rather asymmetric: one side (the “front”, viewed from the H1‐H2 face) is highly positively charged and the other (the “back”) highly negatively charged (Figure 3c). When the same calculation was carried out for the AlphaFold (Jumper et al., 2021) model of HHD1, an asymmetric but less marked pattern was observed (Figure 3d). Although a degree of asymmetry in the charge distribution is found in other HHDs, the difference in RTEL1 seems to be more striking; as none of the other proteins containing an HHD is involved in nucleic acid metabolism, this charged surface may be functional to bind negatively charged nucleic acid (Figure 3c).

The ConSurf server (Yariv et al., 2023) was used to analyze the evolutionary conservation of HHD1 and HHD2 and to calculate the conservation score of each amino acid based on multiple sequence alignments. When the results are associated with a coloring scheme (maroon for highly conserved residues, and turquoise for non‐conserved ones) and projected onto the protein surface, a cluster of conserved positively charged residues is found at the bottom of the molecule, close to the entrance of the H1‐H2 groove (Figure 3c,d and Figure S2).

HHDs are typically involved in intermolecular interactions; although there is a degree of plasticity, the main mode of interaction involves the binding of an amphipathic helix from a partner protein between the H1 and H2 helices of the HHD. This pattern is illustrated by the structure of the HHDs from cerebral cavernous malformations 2 protein (CCM2) (Wang et al., 2015), which either binds in *cis* a sixth C‐terminal helix from the same polypeptide, or an amphipathic helix from other partners, such as the mitogen‐activated protein kinase MEKK3 (Wang et al., 2015) (Figure 3b). Various putative partners have been implicated in binding to the RTEL1 C‐terminal domain. For example, it has been hypothesized that this region binds the tumor suppressor SLX4 by recognizing an amphipathic helix located between SLX4 residues 604–620; (Takedachi et al., 2020) yeast 2 hybrid data suggest that the SLX4 region binds specifically to HHD1 and not HHD2. Indeed, our analysis shows that HHD1 is likely to have a more pronounced groove, and is therefore more likely to bind an amphipathic helix, similarly to the canonical HHDs. Indeed the RTEL1 HHD2 domain has been shown to bind the 32C winged helix domain of RPA; the 3D model based on the NMR chemical shift perturbations suggests that the HDD2 H1‐H2 groove interacts with a RPA‐32C C‐terminal β‐strand and adjacent loops, suggesting that these domains can exhibit a degree of plasticity and show different binding modes from the canonical interaction with an amphipatic helix (Kumar et al., 2024).

### 2.5 SAXS analysis of RTEL1 C‐terminal fragments

To gather structural information on the architecture of the human RTEL1 C‐terminal domain, we carried out Small‐Angle X‐ray Scattering (SAXS) on all the available fragments. SAXS data were collected at BioSAXS beamline P12 (PETRA III, Hamburg, Germany) (Blanchet et al., 2015). To increase the data quality, SEC‐SAXS mode was employed, where a size exclusion chromatography column is connected directly to the SAXS measuring capillary, in order to separate possible oligomers.

Structural parameters such as radius of gyration (*R*_G_) describing the compactness of the sample, the maximum distance (*D*_max_) and molecular weight are summarized in Table 2. The parameters are consistent with the presence in solution of a monomeric form for all of the fragments.

**TABLE 2:** SAXS data collection and primary data analysis.

	HHD1	HHD2	HHD1‐2	CT‐S	CT‐L
(a) Sample details					
Buffer (50 mM)	TRIS	Hepes	Hepes	Hepes	Hepes
pH	8	7	7	7	7
Additives	150 mM NaCl, 10 (v/v) % Glycerol, 1 mM TCEP				
Sample temperature (°C)	20				
In beam sample cell^e^	1 mm quartz capillary				
Organism	Human				
Size exclusion chromatography					
Sample concentration, mg/mL	2.7	6.2	7.2	7.0	3.0
Sample injection volume	40 μL				
SEC column type	Superdex 75 5/100 inc				
SEC flowrate, mL/min	0.2				
(b) SAS data collection					
Data acquisition/reduction software	SASFLOW/Chromixs				
Source/instrument description	EMBL P12, 6M Pilatus				
Measured *s*‐range (s_min_–s_max_; nm^−1^)	0.05–4.4				
Exposure time (s)	0.5 s (~40 frames averaged)				
(c) SAS‐derived structural parameters					
Methods/Software	ATSAS (Primus)				
Guinier analysis					
*R*_G_ ± *σ* (nm)	1.7 ± 0.1	1.5 ± 0.1	3.4 ± 0.1	4.9 ± 0.1	4.9 ± 0.1
Data point range	20–240	45–200	8–119	20–149	14–140
Linear fit assessment (fidelity)	0.88	0.48	0.62	0.36	0.88
PDDF/*p*(*r*) analysis					
*D*_max_ (nm)	4.6 ± 0.2	5.0 ± 0.2	10.2 ± 0.5	18 ± 0.5	15.3 ± 0.5
*p*(*r*) fit assessment (fidelity)	0.88	0.85	0.72	0.73	0.79
(d) Scattering particle size					
Molecular weight (M) estimates (kDa)					
From chemical composition	9.6	10.6	28.3	50.0	58.5
From SAS, Bayesian	9.5 7.9–9.9	8.5 8.0–10.0	31.7 29.9–34.2	72.4 51–80	83.1 75–96
Porod volume (nm^3^)	22 ± 2	24 ± 2	36 ± 3	121 ± 10	132 ± 10
(e) Atomistic modeling methods					
Software	Crysol	Crysol	EOM	EOM	EOM
*χ* ^2^	1.1	1.0	1.0	1.0	1.0
(f) Data and model deposition					
SASBDB IDs	SAS DRZ9	SAS DRX9	SAS DRW9	SAS DRY9	SAS DRZ9

**FIGURE 4:**
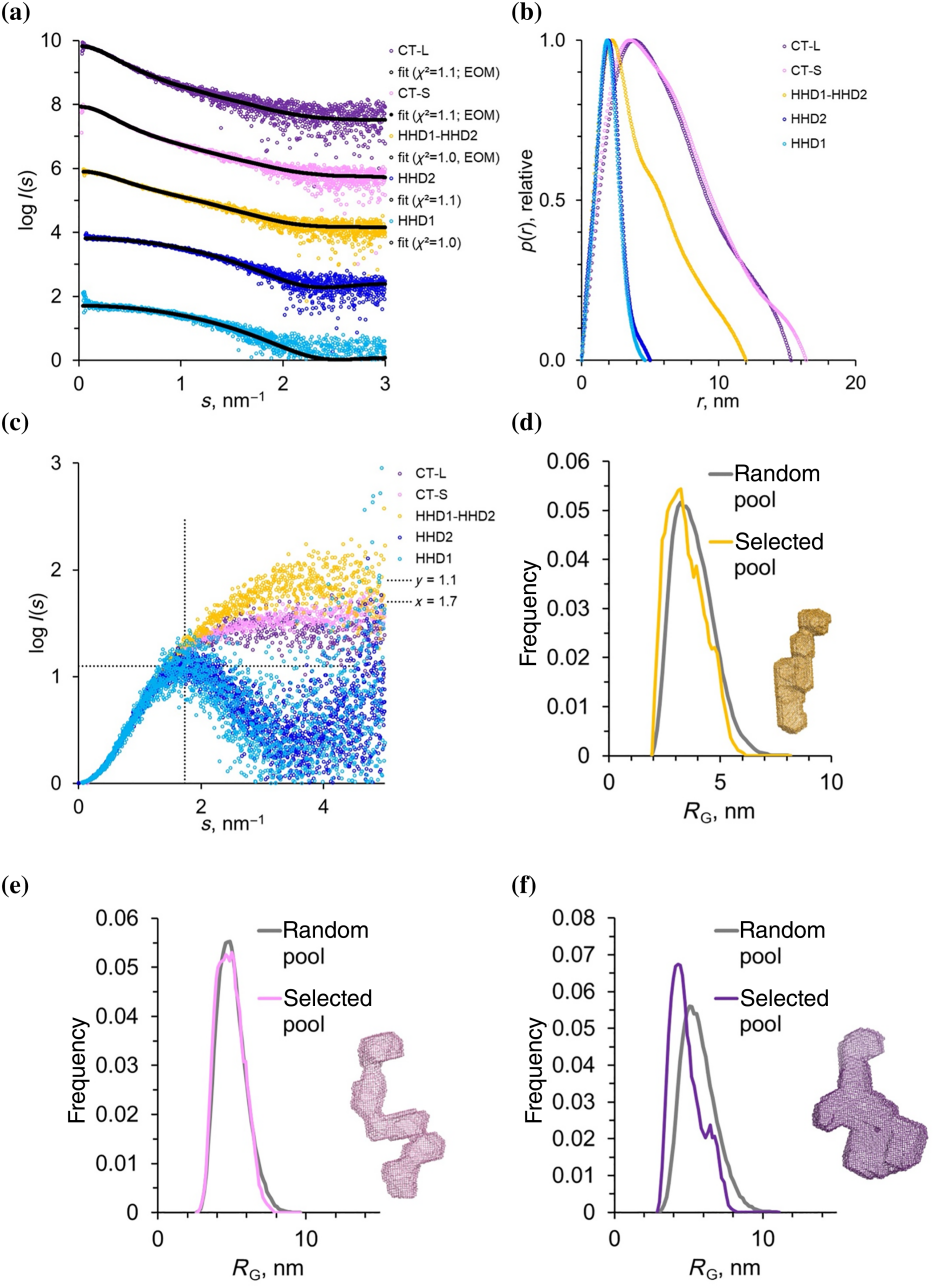
Small‐angle X‐ray Scattering analysis. (a) Experimental SAXS profiles for the C‐terminal fragments in log plot. The profiles have been shifted along the y‐axis for better visibility. The fits from the respective modeling approaches are shown in black and the *χ* ^2^ values are indicated. The HHD1 and HHD2 fragments are fitted with the HHD2 crystal structure using Crysol (Svergun et al., 1995). For HHD1‐HHD2, CT‐S and CT‐L EOM (Tria et al., 2015) fits are shown. (b) the pairwise distance distribution functions *p*(*r*) derived from SAXS data (c) Dimensionless Kratky plots, *sR*_G_^2^ *I*(*s*)/*I*(0) versus *sR*_G_, for direct comparison of flexibility (independent of size). Dotted lines at *sR*_G_ = 1.104, and *SR* ^2^ *I*(*s*)/*I*(0) = √3 are added as an indication of expected peak for globular systems. (d–f) EOM (Tria et al., 2015) analysis and Dammif (Franke & Svergun, 2009) model. The comparison of *R*_G_ distributions for the selected pools to the distribution of the random pools are indicated for HHD1‐HHD2 (d), CT‐S (e) and CT‐L (f). Full EOM (Tria et al., 2015) analysis is shown in Figures S2–S4. Inlets in panels d–f show ab initio envelopes; due to the flexibility of the systems these envelopes do not display an actual state of the constructs but, as averaged structure, they indicate the conformational state occupied by the flexible systems.

The experimental SAXS curves for the HHD1 and HHD2 fragments were in very good agreement with the theoretical scattering profiles computed from the HHD2 X‐ray coordinates (Figure 4a). For these two constructs, the pair‐wise distance distribution, *p*(*r*), functions (Figure 4b), as well as the dimensionless Kratky plots (Figure 4c), indicate that the fragments fold into globular structures.

In the case of the other three fragments, however, the skewed *p*(*r*) functions as well as the plateauing in the Kratky plot suggest more flexible systems. Thus, to gain conformational information on these structures the Ensemble Optimization Method (EOM, Tria et al., 2015) was employed. With EOM the degree of flexibility is assessed by comparing the properties of the selected models (which fit the experimental data) with those of the large pool of randomly generated models. This is done by comparing the distributions of overall parameters such as *R*_G_ and *D*_max_ of the selected pool with the original random pool.

For the region encompassing HHD1 and HHD2 (Figure 4d and Figure S3) these two distributions overlap suggesting that the linker is indeed very flexible and allows the fragment to cover a large conformational space. Similar, for the CT‐S fragment the analysis shows a distribution that almost overlaps with that of the random pool, suggesting that the protein is highly flexible (Figure 4e and Figure S4). The presence of the RING domain in the CT‐L fragment appears to make the overall structure more compact: although still showing a significant degree of flexibility, the system thus appears to assume a more limited number of conformations (Figure 4f and Figure S5).

### 2.6 Biochemical characterization of the human RTEL1 C‐terminus

We then carried out a systematic biochemical analysis of the nucleic acid binding properties of the C‐terminal domain.

**FIGURE 5:**
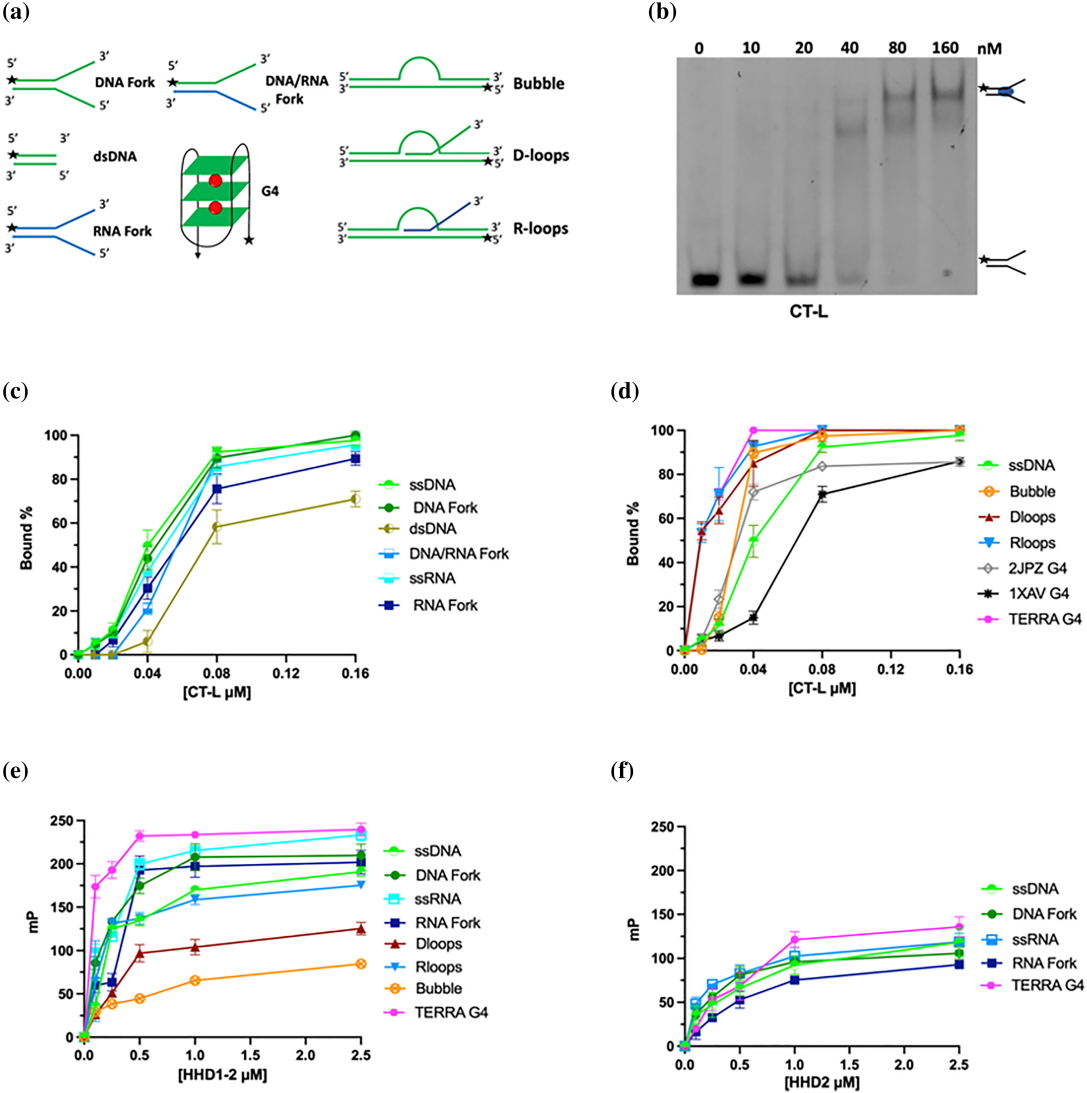
Biochemical affinity of RTEL1 C‐term for a variety of DNA and RNA substrates. (a) A schematic diagram of the substrates used. A star denotes the position of the fluorescent 6‐FAM moiety. (b) Example of a gel shift assays for the CT‐L fragment using a DNA fork. The assays were carried out at increasing concentrations of protein (0–160 nM); each experiment was repeated at least three times. (c) Comparison of CT‐L binding affinity towards a variety of canonical substrates. The protein binds with similar affinity ssDNA, ssRNA, DNA forks, DNA/RNA forks, RNA fork, having a lower affinity for dsDNA. (d) Comparison of the CT‐L affinity towards non‐canonical nucleic acid substrates. The protein shows a preference for TERRA G4 structure, closely followed by R‐loops and D‐loops; DNA G4 structures bind less well. (e) Fluorescence anisotropy measurements for the binding of HHD1‐2 to different substrates. The protein has a significant lower affinity when compared to the whole C‐terminal domain, but a higher degree of specificity. (f) Fluorescence anisotropy measurements for the interactions between HHD2 domain with TERRA G4, ssDNA and ssRNA, DNA and RNA forks. No substrate preference is observed. A binding analysis based on the one‐site specific binding with Hill slope model (GraphPad Prism 10) is reported in Figure S6; the corresponding dissociation constants are listed in Table S3.

The longest fragment, encompassing the whole C‐terminal region (CT‐L), was tested for the ability to bind different oligonucleotide substrates using EMSA with fluorescent substrates. As RTEL1 has been reported to have a key role in homologous recombination, D‐loop metabolism and in counteracting G4/R‐loops‐driven instability, a variety of nucleic acid probes has been tested, including single stranded DNA and RNA, DNA and RNA forks, DNA/RNA hybrid forks, as well as D‐loops, R‐loops and G4 with different topologies (Figure 5a and Table S2). Fluorescent nucleic acid substrates were incubated with increasing protein concentration (0–160 nM), and the mixture was analyzed by polyacrylamide gel electrophoresis to monitor the formation of protein‐nucleic acid complex (Figure 5b). With most substrates two bands appeared in the gel at high protein concentrations, suggesting the presence of complexes with different stoichiometries; although in our hands the CT‐L always behaved as a monomer, either in size exclusion chromatography or in SAXS data (Figure 4), a dimer could form in the presence of nucleic acids.

When canonical nucleic acid substrates were tested, the CT‐L fragment bound with a similar affinity ssDNA, ssRNA, DNA fork, and DNA/RNA hybrids forks, showing a slightly lower affinity for an RNA fork. The affinity for dsDNA was significantly lower, suggesting that CT‐L has a preference for substrates containing a single stranded region (Figure 5c).

In line with the putative cellular roles of RTEL1, we then tested non‐canonical substrates. As previously reported (Ghisays et al., 2021), the C‐terminal domain of RTEL1 shows a high specificity for the RNA G4 found in the TERRA (Telomeric Repeats containing RNA) transcript, reflecting the role of RTEL1 in telomere metabolism (Figure 5d). Two DNA G4 were tested, one found in human telomers (whose structure has been deposited in the Protein Data Bank with ID 2JPZ; Dai et al., 2007), and one present in the promoter of the MYC gene PDB ID: 1XAV (Ambrus et al., 2005). CT‐L has a preference for the telomeric, rather than the promoter G4, but they both bind with a lower affinity than TERRA.

The protein also binds D‐loops and R‐loops significantly better that the canonical substrates and a simple bubble substrate, with a mild preference for R‐loops (Figure 5d). The affinity towards displacement loop structures is comparable to that of TERRA G4, in line with the proposed cellular role of RTEL1 (Björkman et al., 2020; Kotsantis et al., 2020; Vannier et al., 2013; Wu et al., 2020; Youds et al., 2010). Values for the dissociation constants (*K*_d_) for each substrate were calculated using the one‐site specific binding with Hill slope equation (GraphPad Prism 10) and are listed in Table S3, and the corresponding curves shown in Figure S6.

To further dissect the interaction, we also explored the ability of both HHD1‐2 fragment and the single HHD domains to bind different substrates using fluorescence anisotropy (Figure 5e,f). When the single HHD domains were used, a low level of binding was observed for the HHD2 domain (Figure 5f); the binding was clearly unspecific, with most of the substrates tested binding with similar affinity. No binding was observed with HHD1 (data not shown). Despite the fact that the two domains are expected to have a very similar fold, the discrepancy in binding is not surprising, and it is in line with the difference in surface residues and charge distribution (Figure 2c,d), with an asymmetric electrostatic surface charge distribution and a strongly positively charged region in HHD2, likely to underpin the interaction with nucleic acid. This further stresses the divergence and specialization of the two domains, following duplication.

When the region encompassing the two HHD domains and the linker region (HHD1‐2) was tested, the binding affinities were lower than the full‐length protein but clearly significant. When compared to CT‐L, the HHD1‐2 protein displayed a higher degree of specificity towards the different substrates, with TERRA G4 being the best substrate (Figure 5e). A significantly lower affinity was observed for R‐loops, and even more for D‐loop and bubble substrates (Figure 5e). The overall analysis suggests that the HHD1‐2 linker domain has an important role in nucleic acid binding (as HHD1 does not bind and HHD2 has a very low affinity), but that some of the determinants of binding that modulates the recognition of D‐loops and R‐loops are outside this region.

This picture is consistent with the pivotal role which RTEL1 has in the metabolism of G‐rich telomeres ends, folding into G‐quadruplexes, and G4‐associated R‐loops (Kotsantis et al., 2020; Vannier et al., 2012; Wu et al., 2020). The affinity for D‐loops is coherent with the data obtained on recombinant full‐length RTEL1, which was shown to unwind D‐loops in vitro, with a preference for D‐loops with a 3′ invading strand and a free 5′ end (Barber et al., 2008; Youds et al., 2010).

## 3 CONCLUSIONS

We report a comprehensive biochemical and structural analysis of the C‐terminus of human RTEL1, as a framework to help elucidating its multifaceted roles in DNA replication, DNA repair and telomere maintenance. By expressing and purifying a number of fragments encompassing the folded domains and disordered regions, and combining X‐ray crystallography, small‐angle X‐ray scattering and biochemical assays, we provide novel insights into the architecture and function of this region.

The crystal structure of the second C‐terminal repeats confirms that it folds as an harmonin homology repeat (Figure 3a). Whereas HHD domains tend to bind amphipathic helices in the groove between helices H1 and H2, the RTEL1 HHD2 domain has a shallower groove; within the crystal lattice, an helix from an adjacent molecule binds to the groove with a different orientation with respect to the canonical HHD recognition, suggesting the possibility of a slightly different mode of binding (Figure 3b). Small‐angle X‐ray scattering provides low resolution information on the different regions; SAXS data suggest that the presence of the RING domain has a significant impact on the overall C‐terminal architecture: whether the longer fragments lacking the RING domain are extremely flexible, the presence of the RING domain makes the protein relatively more compact.

In addition to the well‐studied PIP box located between the HHD2 and the RING domain (PIP1), we identified a second putative PIP box (PIP2) at the very C‐terminal end of the protein (Figure 1a); we measured the binding of both PIP boxes to PCNA, using a combination of NMR chemical shift perturbation and isothermal calorimetry (Figure 2). Although we see a significantly stronger binding to PCNA for the well‐established PIP1 box, PIP2 gives a weaker signal that is still located in the PIP‐binding region of PCNA, and may thus act as ancillary PIP box, acting in a dynamic network of interactions, as seen in a number of enzymes involved in DNA metabolism (Hamdan & De Biasio, 2023).

Biochemical assays performed with the entire C‐terminus and its fragments provide valuable insights into RTEL1 nucleic acid binding preferences (Figure 5). In addition to confirming the binding affinity towards RNA and DNA G‐quadruplex structures found at telomers, we measure a high affinity for both D‐loops and R‐loops, which is consistent with RTEL1's crucial role in maintaining telomere stability and resolving G4/R‐loops structures to prevent transcription‐replication clashes (Barber et al., 2008; Ghisays et al., 2021; Kotsantis et al., 2020; Vannier et al., 2012; Wu et al., 2020; Youds et al., 2010). Moreover, by analyzing the interactions between each of the purified fragments, encompassing a number of folded domains and connecting links, and a panel of nucleic acids, we can dissect the role of each region. For example we find that whereas HHD1 has no binding activity, HHD2 shows a weak and unspecific binding, in line with the strong positively charged region on one face of the domain; when the region encompassing both HHD1 and HHD2 and the connecting link is tested, the protein has a significant affinity, with a distinct specificity for TERRA and a much weaker binding to D‐loops and R‐loops, indicating that the determinants for displacement loops binding are outside this region.

These results thus provides a useful framework for further studies leading to better understand the structural determinant of the interaction between the RTEL1 C‐terminal region and protein partners (including SLX4, PCNA and RPA) as well as the structural basis of binding to nucleic acid substrates; they can also guide cell biology studies that aim to use mutated/truncated versions of the C‐terminal region.

## 4 MATERIALS AND METHODS

### 4.1 Bioinformatic analysis

Database searches were carried out using the BLAST algorithm (Altschul et al., 1990) multiple sequence alignments were generated with the Clustal Omega program and manually modified to account for the positioning of the secondary structure elements and the novel features emerging from the fold recognition analysis. A variety of threading/fold recognition algorithms were also used to identify or confirm the presence of structural features. These include the structure prediction server RaptorX (Källberg et al., 2012), HHpred (Gabler et al., 2020), FFSA03 (Jaroszewski et al., 2005) and AlphaFold (Jumper et al., 2021). The level of conservation for HHD1 and HHD2 was calculated using the ConSurf web server (https://consurf.tau.ac.il/); to discriminate between HHD1 and HHD2 sequence alignments were manually provided (Yariv et al., 2023).

### 4.2 Cloning

hRTEL1 constructs were cloned into a pNIC‐TrxT vector, a gift from Opher Gileadi, Structural Genomics Consortium, Oxford (Savitsky et al., 2010) with the LIC method (Aslanidis et al., 1994), using the cDNA corresponding to the human hRTEL1 isoform 3 as a template. All constructs included an N‐terminal 6His‐Thioredoxin tag followed by a TEV protease cleavage site. A list of all the primers used for cloning is found in Table S1.

### 4.3 Protein expression and purification

Uniformly ^2^H,^13^C,^15^N‐PCNA for NMR experiments were prepared as described (Sánchez et al., 2007). Briefly, human PCNA (UniProt: P12004) was produced in *Escherichia coli* BL21(DE3) Rosetta cells (grown in isotopically enriched medium) with an N‐terminal 6His‐tag and a HRV 3C protease cleavage site. The protein was purified from the soluble fraction by Co^2+^ affinity chromatography, proteolyzed, and the flow through of a second Co^2+^ affinity chromatography was polished by size exclusion chromatography. The pure protein contained a 3‐residue N‐terminal extension (GPH) preceding the native initial methionine.

All the hRTEL‐1 fragments were expressed in LB media in the *E. coli* B834 (DE3) strain (Novagen). Cells were grown at 37°C to an OD_600_ of 0.7 and then transferred to 18°C. Recombinant protein expression was induced by the addition of 0.1 mM IPTG. After 20 h, cells were harvested by centrifugation. Cell pellets were thawed and resuspended in Lysis Buffer (50 mM Hepes pH 7.5, 500 mM NaCl, 2.5 mM MgCl_2_, 5 mM Imidazole, 10% Glycerol, 1 mM TCEP) supplemented with 0.1 mg/mL DNase I, 1 mM PMSF, 15 μg/mL Benzamidin, 4 μg/mL Leupeptin and 2 μg/mL Aprotinin. After cells disruption with an EmulsiFlex® homogenizer, the crude extract was centrifuged for 1 h at 30,000 *g* and the soluble fraction was incubated with 1 mL of Ni^2+^ affinity resin (Qiagen). After 1 h of incubation at 4°C, the resin was washed with 20 resin volume of Washing Buffer (Lysis Buffer supplemented with 15 mM imidazole) and the protein was then eluted with the addition of 300 mM imidazole. The N‐terminal fusion tag was cleaved by the addition of 1:50 mass ratio of His‐tagged TEV‐protease during overnight dialysis into TEV Buffer (50 mM Tris–HCl pH 7.5, 150 mM NaCl, 0.5 mM EDTA, 1 mM DTT, 10% Glycerol). After TEV removal, the flow‐through fraction, containing the protein, was collected and the salt was reduced to 50 mM before applying to a HiTrapSP HP column (Cytiva). The column was washed with 10 CV (Column Volume) of Cation buffer (50 mM Hepes pH 7.5, 50 mM NaCl, 10% Glycerol, 0.5 m TCEP) and the protein was eluted with a linear gradient over 40 CV to a concentration of 1 M NaCl. For HHD1 fragment, an anion exchange was used and the protein was applied to a HiTrapQ HP column (Cytiva) which has been previously pre‐equilibrated with Anion Buffer (50 mM Tris–HCl pH 8.0, 10% Glycerol, 50 mM NaCl, 0.5 mM TCEP). As a final polishing step, all RTEL‐1 fragments were loaded onto an analytical Superdex 75 Increase 10/300GL size exclusion column, using protein‐storage buffer (50 mM Hepes pH 7.5, 150 mM NaCl, 10% Glycerol, 1 mM TCEP). Proteins were finally flash frozen with liquid nitrogen and stored at −80°C.

### 4.4 Oligonucleotide preparation

All the oligonucleotides were chemically synthesized and purified by reverse‐phase high pressure liquid chromatography and PAGE (Biomers.net). Selected oligos were labeled with the fluorescent dye 6‐Carboxyfluorescein (6‐FAM). DNA and RNA oligos (Table S2) were resuspended in Tris–EDTA buffer (10 mM Tris–HCl pH 7.5, 1 mM EDTA) to a concentration of 100 μM and stored at −20°C. G4‐oligos were instead resuspended in potassium phosphate buffer (10 mM KH_2_PO_4_/K_2_HPO_4_, 50 mM KCl, at pH 7.5). The RNase inhibitor RNase OUT (Thermo Fisher Scientific) was added to reactions containing RNA substrates (0.16 units/μL, 1:250).

For EMSA assays, the substrates used were prepared using the following protocols:

For *fork‐DNA* (D1L:D3L), *dsDNA* (D1B:D3B), *fork‐RNA* (R1L:R3L), *dsRNA* (R1B:R3B), *hybrid fork‐DNA/RNA* (D1L:R3L) and *bubble* (D9:D11) the fluorescent strand and the complementary strand were annealed at a 1:3 molar ratio in 20 mM Tris pH 8.0 and 100 mM NaCl. 100 μL reactions including 10 μM of each strand were heated to 95°C for 5 min and gradually cooled to 15°C at a rate of 1°C/min using a PCR thermal cycler (Eppendorf). After annealing, the G4 substrates were further incubated at 4°C overnight.

For *D‐loop* (D4:D9:D11) and *R‐loop* (R4:D9:D11) the molar ratio used were 1:1.25:2.5 in 6 mM Tris–HCl pH 7.5, 7 mM MgCl_2_, 50 mM NaCl and 1 mM DTT. The annealing method used for D‐loop and R‐loop was heating at 99°C for 5 min followed by incubations at 67°C for 1 h, at 37°C for 30 min and at 25°C 3–4 h or overnight (Chakraborty & Grosse, 2011). Following electrophoresis in a native 10% acrylamide (29:1) TBE gel at RT in 1X TBE buffer, substrates were eluted from gel slices by dialysis in TE buffer.

### 4.5 Nucleic acid binding assays

Fluorescence anisotropy experiments were carried out in triplicate in a 384‐well plate (Corning, 384‐well, black, low volume, flat bottom, non‐binding surface) at 25°C and fluorescence monitored by a microplate reader (Tecan Infinite F200 PRO) using a 495 nm excitation wavelength and a 525 nm emission wavelength. Every replicate contained a 30 μL solution containing 20 mM Tris pH 7.5, 50 mM KCl, 0.5 mM MgCl_2_, 10% Glycerol, and 0.1% Igepal with 10 nM substrates and increasing concentrations (0–2500 nM) of the purified proteins.

For EMSA, binding reactions were performed in 20 mM Tris pH 7.5, 50 mM KCl, 0.5 mM MgCl_2_, 10% Glycerol, and 0.1% Igepal with 10 nM substrates and increasing concentrations (0–160 nM) of the purified protein in 20 μL of reaction volume, incubated at room temperature for 15 min. The reaction mixture was then loaded onto an 8% non‐denaturing polyacrylamide gel and run at RT in TBE buffer. For the G4 oligos, 10 mM KCl was added to the gel. Fluorescent labeled substrates were detected by fluorescent scanner (ImageQuant, GE Healthcare) and quantification of protein bound nucleic acid was performed with ImageQuant image analysis software (GE Healthcare). Each experiment has been repeated three times and plotted using Prism 8 (Graphpad).

To derive the dissociation constants reported in Table S3 the data was analyzed using the one‐site specific binding with Hill slope equation in GraphPad Prism 10 (Figure S6).

### 4.6 Crystallization and structure determination

Crystals of HHD2 (residues 1053–1147) were grown at 10 mg/mL using the hanging‐drop vapor diffusion technique, in 0.1 M CHES pH 9.5, 30% PEG 3000 at 20°C. Several crystals were harvested, mounted on cryo loops and flash‐cooled in liquid nitrogen with mother liquor with no cryo‐protectant. The crystals were tested at the XRD2 (11.2R) beamline at Elettra‐Sincrotrone Trieste, Italy (Lausi et al., 2015). Diffraction data were collected from a crystal of dimensions ~100 μm × 5 μm × 5 μm which diffracted to about 2.3 Å. Data were collected at 100 K and a wavelength of 1 Å, using a Dectris Pilatus 6 M detector. Data were integrated and scaled using XDS (Kabsch, 2010). Based on systematic absences the protein crystallized in space group P2_1_. Cell content analysis suggests seven molecules in the asymmetric unit.

The coordinates of the human RTEL1 AlphaFold (Jumper et al., 2021) model corresponding to HHD2 (residues 1053–1147) were used for molecular replacement using the program MOLREP (Vagin & Teplyakov, 1997). As the initial attempt showed clashes in the crystal packing, a trimmed hRTEL1 model, from residues 1071 to 1135 was used for molecular replacement. The initial coordinates obtained from the program MOLEP was subjected to 20 cycles of rigid body refinement followed by 10 cycles of restrained refinement using REFMAC5 (Murshudov et al., 2011). To remove any possible model bias the resulting map after restrained refinement was input to the program Buccaneer (Cowtan, 2006) for model building. The programs MOLREP, REFMAC5 and Buccaneer are available as part of the CCP4 software suite (Collaborative Computational Project, 1994). Buccaneer was able to build most of the residues in all the seven molecules. The model was improved by several iterations of model building followed by restrained refinement with REFMAC5, and was further subjected to TLS and restrained refinement. The progress of refinement was monitored by calculation of *R*_free_ using 5% of independent reflections. Table 1 summarizes the data and refinement statistics.

### 4.7 SAXS data collection and analysis

Synchrotron SAXS data from the RTEL1 C‐terminal fragments were collected on the EMBL P12 beamline (Blanchet et al., 2015) at PETRA III (DESY, Hamburg, Germany), using a Pilatus 6M detector at a sample‐detector distance of 4 m and at a wavelength of *λ* = 0.155 nm. Data were collected in static mode (batch), as well as with an integrated SEC purification step, employing in‐line size‐exclusion chromatography. For this, an Agilent 1260 Infinity II Bio‐inert LC, equipped with a GE Superdex 75 Increase 5/150 size exclusion column was used. 50 μL of sample were injected at a flow rate of 0.20 mL/min at 20°C. The column elute was directly streamed to the SAXS capillary cell. Approximately 1200 successive 0.5 s frames were collected. The data were normalized to the intensity of the transmitted beam and radially averaged, resulting in (*I*(*s*) vs. *s* scattering profiles, where *s* = 4*π*sin*θ*/*λ* (momentum transfer), and 2*θ* is the scattering angle. The program CHROMIXS (Panjkovich & Svergun, 2018) was employed for visualization and reduction (background subtraction), both automatically and interactively, of the SEC‐SAXS datasets. The PRIMUS (Konarev et al., 2003) module of the ATSAS software package (Franke et al., 2017) was used for further processing. Forward scattering (*I*(0)) and radius of gyration (*R*_G_) were obtained by fitting the linear Guinier region of the data. Pair distribution function, *p*(*r*), with the corresponding maximum particle size parameter, *D*_max_), was determined using the program GNOM (Svergun, 1992). Theoretical scattering profiles were computed from X‐ray coordinates using Crysol (Svergun et al., 1995). For the reconstruction of a theoretical molecular envelope, ab initio modeling, the program DAMMIF (Franke & Svergun, 2009) was used, where scattering from the calculated envelopes was fitted against the experimental scattering and evaluated by the *χ* ^2^ values. Twenty ab initio models were averaged by using DAMAVER (Volkov & Svergun, 2003). For advance hybrid modeling, ensemble modeling was performed with EOM (Ensemble Optimization Method 2.1, Tria et al., 2015). EOM was run to compare the flexibility of the different C‐terminal constructs. For the HHD1‐2 region, 10,000 random models were generated by using the crystallographic HHD2 structure for both HHD1 and HHD2, connected by a flexible 100 amino‐acid linker. For the initial pool of random models of CT‐S the model was extended by the missing N‐ and C‐terminal residues. The models for CT‐L include the crystal structure of the RING finger domain. The distributions of the *R*_G_ and *D*_max_ for the initial pool of random models are compared to the distribution of the subset of selected models.

The SAXS data are summarized in Table 2 and the respective curves and models have been deposited in the Small Angle Scattering Biological Data Bank (SASBDB; Kikhney et al., 2020).

### 4.8 Peptide synthesis and preparation

The peptides corresponding to the PIP1 and putative PIP2 of hRTEL1 (Figure 2a) were synthesized by the CRIBI Center (University of Padua) on an Applied Biosystem machine, purified by reverse phase HPLC (RP‐HPLC) to a degree of purity higher than 95% as confirmed by MALDI Tof‐Tof analysis. These peptides were being used for structural characterization of the interaction with PCNA using Nuclear Magnetic Resonance (NMR). Both peptides were dissolved in PBS (10 mM phosphate, 140 mM chloride, 153 mM sodium, and 4.5 mM potassium) and pH was adjusted to 7.0. Peptide‐PCNA complexes were prepared by mixing peptide and protein stocks to obtain a molar ratio of 11.5:1 for PIP‐1 and 13.2:1 for PIP‐2.

### 4.9 Nuclear magnetic resonance

NMR experiments were carried out on a Bruker AVANCE III 800 MHz spectrometer equipped with a TCI cryo‐probe and z‐gradients. ^1^H‐^15^N BEST‐TROSY spectra were recorded at 35°C for 12 h on 400 μL samples containing 51 μM PCNA (protomer concentration) in PBS (10 mM phosphate, 140 mM chloride, 153 mM sodium, and 4.5 mM potassium), pH 7.0, 20 μM DSS (4,4‐dimethyl‐4‐silapentane‐1‐sulfonic acid), 0.01% NaN_3_, 1 mM DTT, and 5% ^2^H_2_O in 5 mm shigemi NMR tubes (without plunger). Spectra were recorded in the absence and in the presence of 584 μM PIP1 or 673 μM PIP2 peptide. NMR data were handled and processed using TopSpin v4.1 (Bruker), the analysis was carried out through the CcpNmr Analysis v2 software (Vranken et al., 2005), and the structures were visualized with Pymol (Schrodinger, 2015). The chemical shift perturbations (CSP) of the PCNA signals caused by RTEL1 peptides were computed as the weighted average distance between the backbone amide ^1^H and ^15^N chemical shifts in the bound and free states, as described (De Biasio et al., 2012), and the estimated error in the calculated CSP is ±0.005 ppm.

### 4.10 ITC experiments

Measurements were performed on a MicroCal PEAQ‐ITC calorimeter (Malvern) at 35°C in PBS pH 7.0, 1 mM TCEP. The protein and the PIP1 peptide were separately dialyzed against the same buffer. The sample cell contained 16.8 μM PCNA and the syringe contained 282 μM peptide. The experiment consisted of a series of 19 injections of 2 μL (except the first one of 0.4 μL) with a 150 s delay and stirring at 750 rpm. A dilution experiment was recorded with the same series of injections on a sample containing buffer only. The electrical power required to maintain the cell at constant temperature after each injection was recorded as a function of time, generating the corresponding thermograms. Data were fitted to a 1:1 binding model using the MicroCal PEAQ‐ITC software (Malvern). The best fitting was obtained with 0.92 ± 0.12 number of sites. A control experiment injecting the peptide into the cell with buffer and no PCNA was also performed.

## AUTHOR CONTRIBUTIONS

**Giuseppe Cortone:** Methodology; investigation; formal analysis. **Melissa A. Graewert:** Methodology; data curation; formal analysis; supervision. **Manil Kanade:** Methodology; data curation. **Antonio Longo:** Methodology; data curation. **Raghurama Hegde:** Data curation; methodology. **Amaia González‐Magaña:** Methodology. **Belén Chaves‐Arquero:** Methodology. **Francisco J. Blanco:** Methodology; formal analysis; supervision. **Luisa M. R. Napolitano:** Conceptualization; investigation; writing – original draft; supervision. **Silvia Onesti:** Conceptualization; investigation; funding acquisition; writing – original draft; supervision.

## FUNDING INFORMATION

This work was supported by the Fondazione Italiana per la Ricerca sul Cancro, through grant IG 20778. The project also received funds from the European Union's Horizon 2020 Research and Innovation Programme under the Marie Sklodowska‐Curie grant agreement no. 859853 (AntiHelix). SAXS data collection has been supported by iNEXT‐Discovery, grant number 871037, funded by the Horizon 2020 program of the European Commission. Work at CIB‐CSIC has been supported by MCIN/AEI/10.13039/501100011033 (PID2020‐113225GB‐I00 to FJB). AL gratefully acknowledges CERIC‐ERIC for funding within the framework of INTEGRA and INTEGRA's PhD project.

## CONFLICT OF INTEREST STATEMENT

The authors declare that they have no conflict of interest.

## Supporting information


Appendix S1

## Data Availability

Coordinates and structure factors for the HHD2 crystal structure have been deposited to the Protein Data Bank, under accession code 8P8H. For all the SAXS structure presented herein, coordinates and structure factors have been deposited to the Small Angle Scattering Biological Data Bank (SASBDB) under accession codes SASDRV9 (CTL), SASDRX9 (CTS), SASDRZ9 (HHD1), SASDRY9 (HHD2), SASDRW9 (HHD1‐HHD2).
