# Supplementary material for: Structural and biochemical characterization of the C‐terminal region of the human RTEL1 helicase
Source: Protein Sci. 2024 Aug 24;33(9):e5093. doi: 10.1002/pro.5093 (PMC11344278; doi:10.1002/pro.5093)
Supplement: Supplementary file 1 — Appendix S1 [file PRO-33-e5093-s001.docx]

*Supporting Information for*

**Structural and biochemical characterization of human RTEL1 C-terminal domain**

Giuseppe Cortone, Melissa A Graewert, Manil Kanade, Antonio Longo, Raghurama Hedge, Amaia González-Magaña, Belén Chaves-Arquero, Francisco J Blanco, Luisa MR Napolitano and Silvia Onesti.

**Table S1**

| **Fragment** | **Forward (5’-3’)** | **Reverse (5’-3’)** |
| --- | --- | --- |
| **CT-L** | ATGCCAGCGCCGGCC | CTGGGGCTCTGGCCA |
| **CT-S** | ATGCCAGCGCCGGCC | CTCACCCCACTCAGA |
| **HHD1-2** | GTGGCTGGTGCACAG | TCCCGGTGGCTCCAT |
| **HHD1** | GTGGCTGGTGCACAG | TCGTCCTGTCAGCTG |
| **HHD2** | GGGAAGCAGGGCCAG | TCCCGGTGGCTCCAT |

***List of primers used for the cloning of human RTEL1 C-terminal fragments.*** *The following sequences complementary to the vectors have been added to the 5’ end of primers: forward ^5’^TACTTCCAATCCATG^3’^ (ATG in frame with the desired coding sequence), reverse ^5’^TATCCACCTTTACTGTCA^3’^.*

**Table S2**

| Name | nucleotide sequence (5’-3’) |
| --- | --- |
| *D1* | [6-FAM]-CTACTACCCCCACCCTCACAACCTTTTTTTTTTTTTTT |
| *D1B* | [6-FAM]-CTACTACCCCCACCCTCACAACC |
| *D3* | TTTTTTTTTTTTTTTGGTTGTGAGGGTGGGGGTAGTAG |
| *D3B* | GGTTGTGAGGGTGGGGGTAGTAG |
| *R1* | [6-FAM]-cuacccccacccucacaaccuuuuuuuuuuuuuuu |
| *R3* | uuuuuuuuuuuuuuugguugugagggugggggua |
| *D11* | GTTGTAAAACGACGGCCAGTGCCTTTTCCCAGCCTCAATCTCATCACTCTAGAGGATCCCCGGGTAC |
| *D9* | [6-FAM]-GTACCCGGGGATCCTCTAGAGTCGACCTGCAGGCATGCAAGCTTGGCACTGGCC GTCGTTTTACAAC |
| *D4* | GCTTGCATGCCTGCAGGCCAGCCTCAATCTCATC |
| *R4* | gcuugcaugccugcaggccagccucaaucucaucuuuuu |
| *2JPZ* | [6-FAM]-TTAGGGTTAGGGTTAGGGTTAGGGTT |
| *1XAV* | [6-FAM]-TGAGGGTGGGTAGGGTGGGTAA |
| *TERRA* | [6-FAM]-uuaggguuaggguuaggguuaggg |

***List of the oligonucleotides for the preparation of the nucleic acid substrates.*** *Names and sequences of the oligonucleotides used to generate the substrates used in the biochemical assays. DNA oligonucleotides are shown in uppercase, RNA oligonucleotides in lowercase. Selected oligonucleotides were labelled with the fluorescent dye 6-Carboxyfluorescein (6-FAM).*

**Table S3**

|  | CT-L | HHD1-2 | HHD2 |
| --- | --- | --- | --- |
| Substrate | **Binding K_d_  (nM)** | **Binding K_d_  (nM)** | **Binding K_d_  (nM)** |
| *ssDNA* | 40 ± 2 | 247 ± 50 | 684 ± 150 |
| *DNA Fork* | 43 ± 2 | 155 ± 30 | 254 ± 52 |
| *dsDNA* | 63 ± 5 | N/D | N/D |
| *DNA/RNA Fork* | 52 ± 3 | N/D | N/D |
| *ssRNA* | 46 ± 2 | 192 ± 72 | 235 ± 76 |
| *RNA Fork* | 51 ± 3 | 273 ± 72 | 512 ± 104 |
| *Bubble* | 27 ± 2 | NA | N/D |
| *D-loops* | 11 ± 2 | 327± 65 | N/D |
| *R-loops* | 10 ± 2 | 160 ± 55 | N/D |
| *2JPZ (G4 telomeric)* | 26 ± 2 | N/D | N/D |
| *1XAV (c-Myc promoter)* | 57 ± 8 | N/D | N/D |
| *TERRA* | 10 ± 2 | 38 ± 18 | 489 ± 124 |
|  |  |  |  |

***Equilibrium dissociation constants (K_d_) for CT-L, HHD1-2 and HHD2 domains.*** *The K_d_ have been determined by fitting the data to an equation representing one site-specific binding with Hill slope equation in GraphPad Prism 10. N/D, not determined; NA, not applicable.*


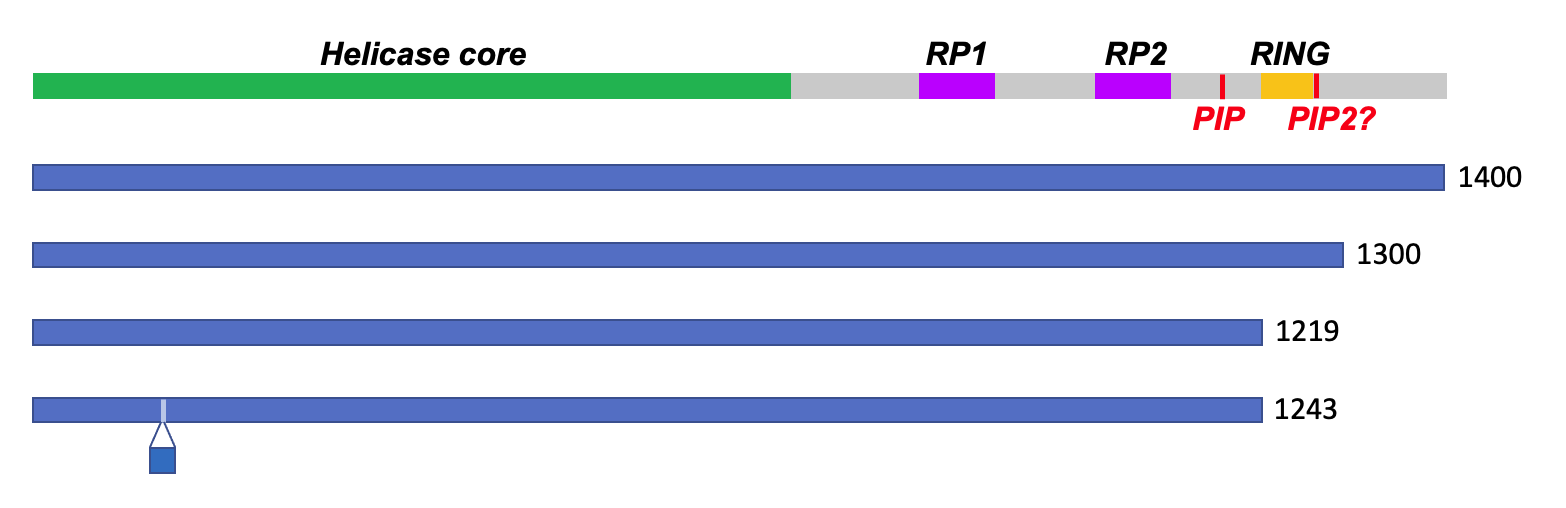


***Figure S1. A schematic diagram of the principal RTEL1 isoforms present in human cells****: the second (1-1300) and third (1-1219) isoforms are the most common. The top diagram reports the domain structure, including the helicase core (in green), the two repeats (in magenta), the RING domain (in yellow), and the position of the well-studied PIP-box, as well as a putative PIP box following the RING domain. The RING domain is only present in the longer isoforms. One isoform (1243) has a 24 amino acid insertion within the helicase core. The 1400 isoform is a read-through transcript into the adjacent TNFRSF6B gene and is likely to be degraded by nonsense-mediated mRNA decay.*

**
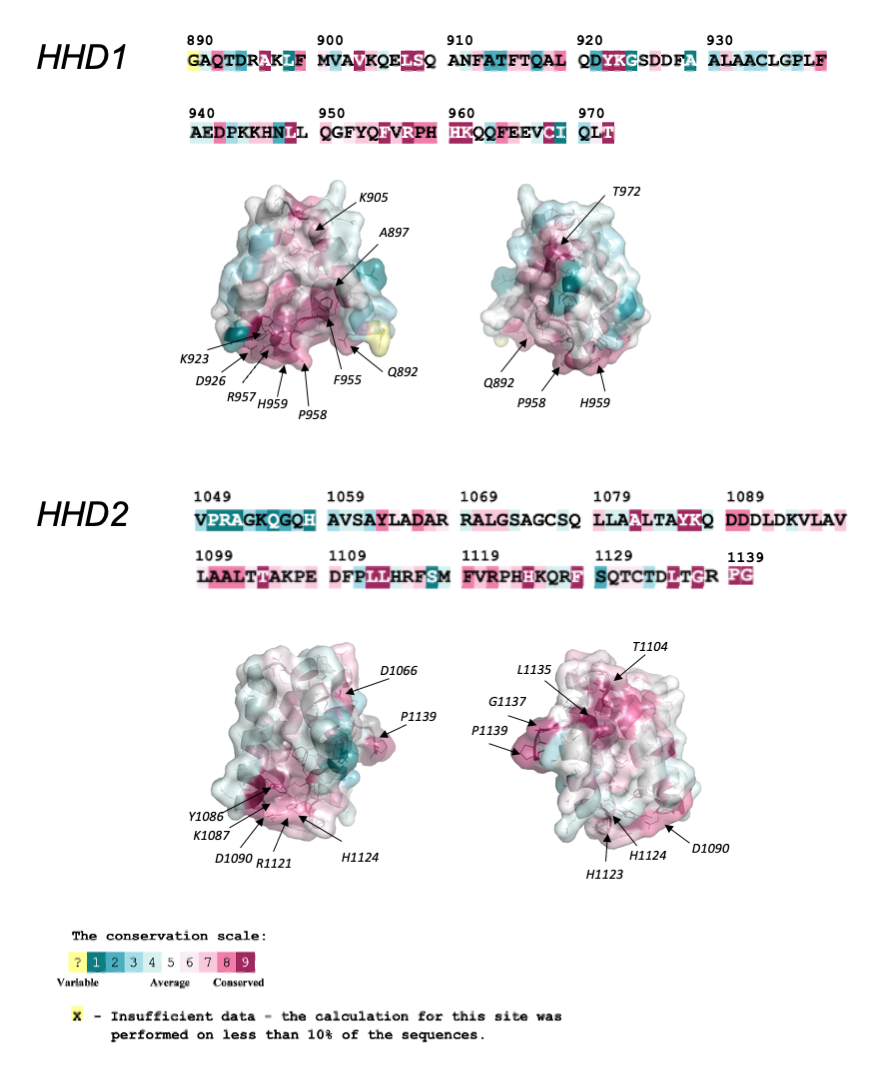
**

***Figure S2. Sequence conservation for the HHD1 and HHD2 domain****. The level of conservation for HDD1 and HDD2 was calculated using the ConSurf web server (https://consurf.tau.ac.il/); to clearly distinguish surface conservation for HHD1 and HHD2, manually curated sequence alignments were used as inputs. The level of surface conservation is displayed so that highly conserved residues are in maroon, and non-conserved residues in turquoise. The position of conserved residues located on the surface are highlighted.*

**
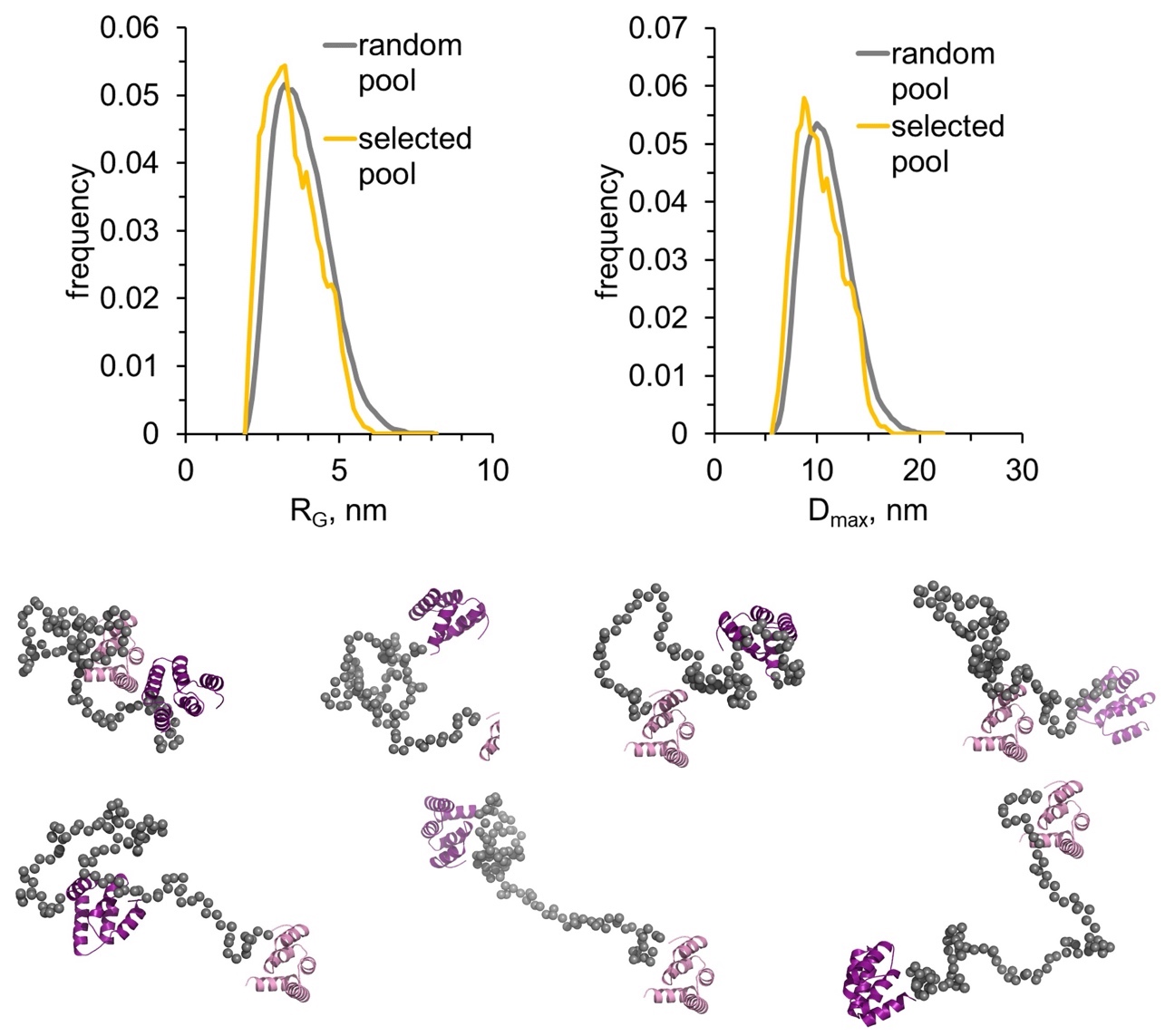
**

***Figure S3. Full EOM-analysis for the HHD1-HHD2 fragment.*** *Rg (left panel) and Dmax (right panel) distributions for a pool (grey) and selected (yellow) ensembles generated by EOM analysis. On the bottom, a representative gallery of bead models for HHD1-HHD2 fragment is shown; the harmonin homology domains HHD1 and HHD2 are shown in pink and violet, respectively.*


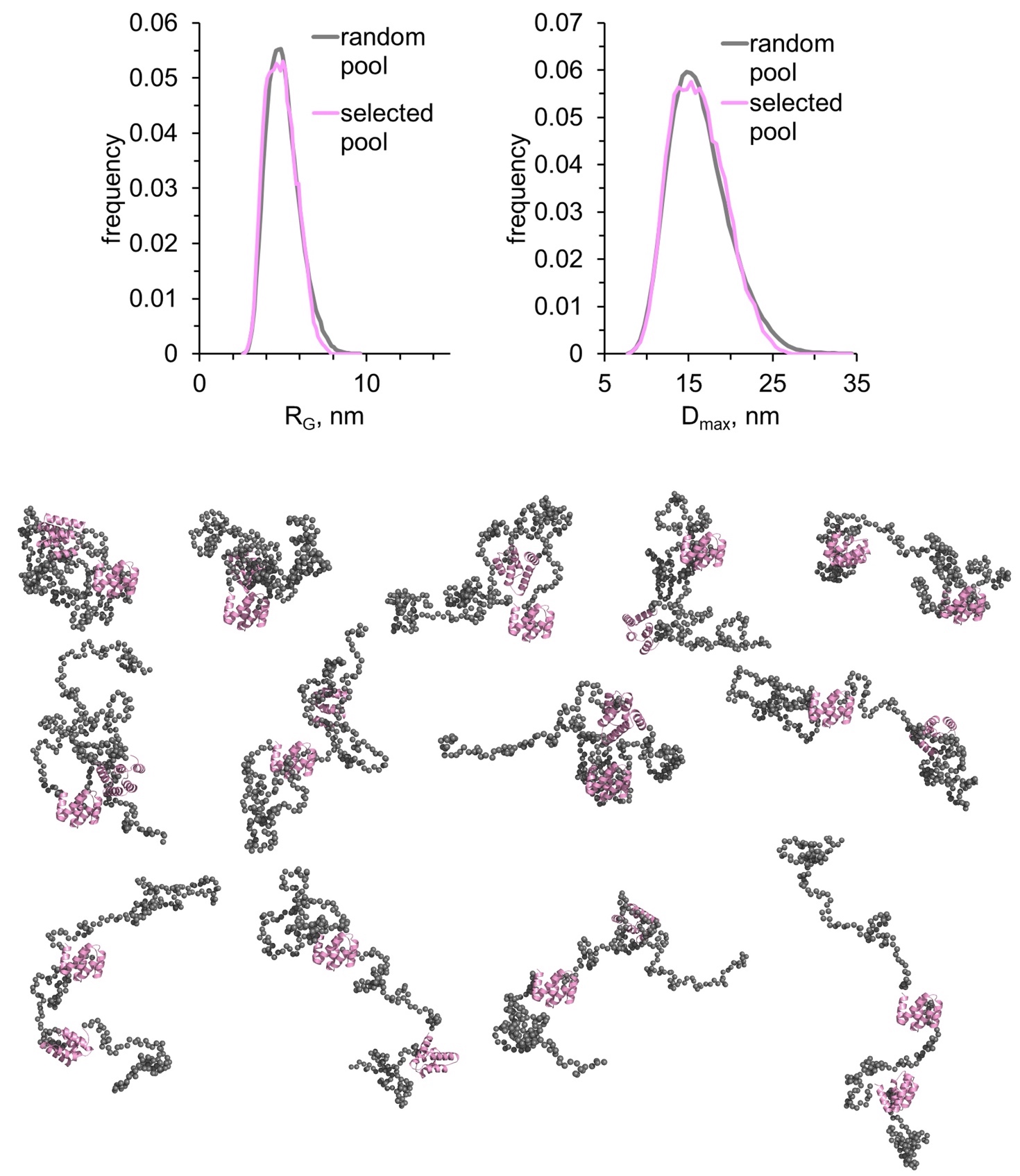


***Figure S4. Full EOM analysis for CTS fragment.*** *Rg (left panel) and Dmax (right panel) distributions for a pool (grey) and selected (pink) ensembles generated by EOM analysis. On the bottom, a representative gallery of bead models for CTS fragment is shown; HHD1 and HHD2 are shown in pink.*

**
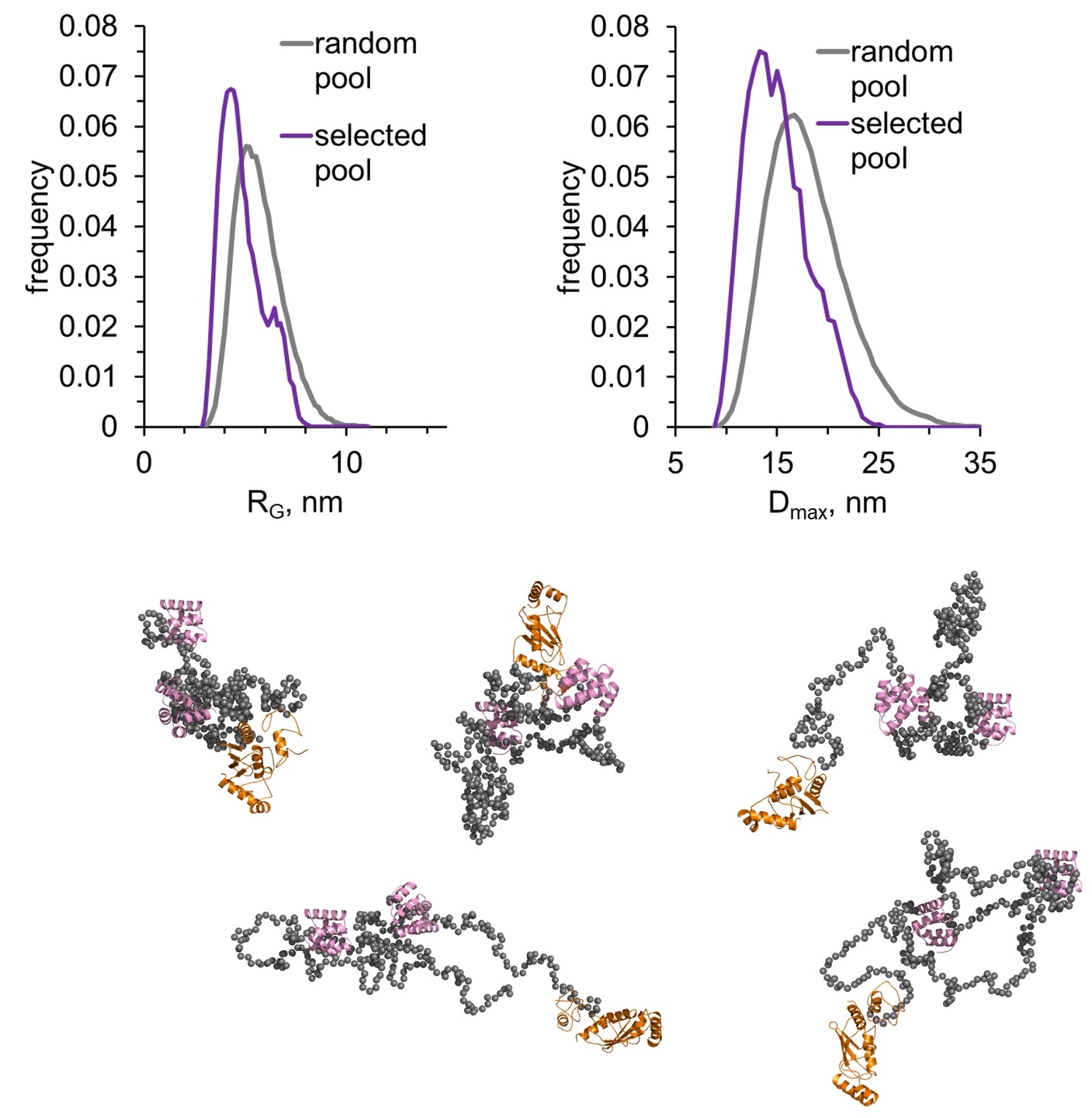
**

***Figure S5. Full EOM analysis for CTL fragment.*** *Rg (left panel) and Dmax (right panel) distributions for a pool (grey) and selected (violet) ensembles generated by EOM analysis. On the bottom, a representative gallery of bead models for CTL fragment is shown; HHD1 and HHD2 are shown in pink, whereas the RING domain is shown in orange.*


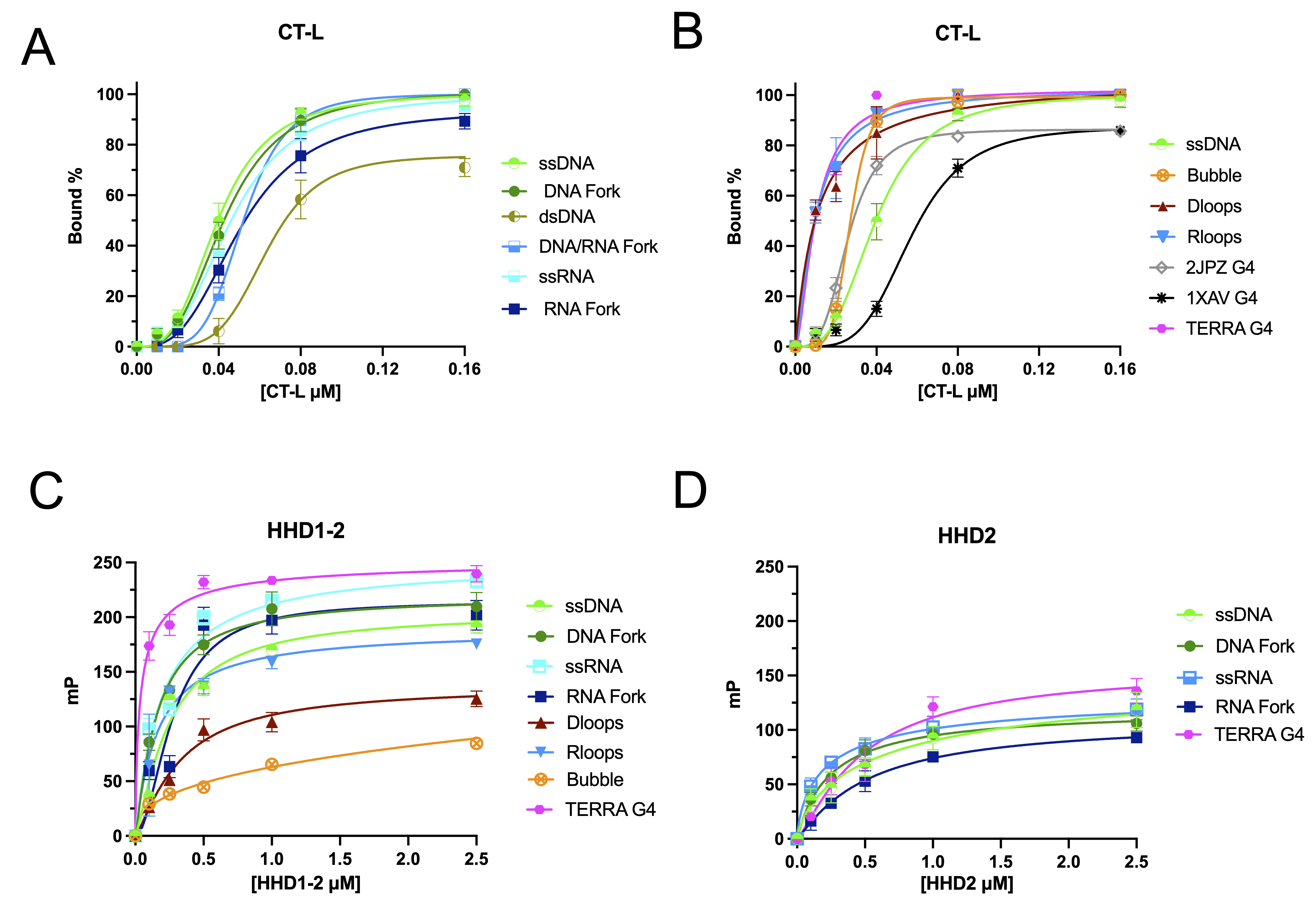


***Figure S6. Binding analysis of RTEL1-C-term for a variety of DNA and RNA substrates.*** *Graphs of fraction bound plotted against protein concentration analysed by one-site specific binding with Hill slope model in GraphPad Prism 10, n= 3 experimental repeats, mean and standard deviation delimited by error bars shown. The corresponding binding constants are reported in Table S3.* ***A)*** *CT-L binding affinity* *towards a variety of canonical substrates, based on Electrophoretic Mobility Shift Assays (EMSA).* ***B)***  *CT-L affinity towards non-canonical nucleic acid substrates, based on EMS).* ***C)*** *Fluorescence anisotropy measurements for the binding of HHD1-2 to different substrates.* ***D)*** *Fluorescence anisotropy measurements for the interactions between HHD2 domain with TERRA G4, ssDNA and ssRNA, DNA and RNA forks.*
